# The Clinical Course of Early and Late Mild Cognitive Impairment

**DOI:** 10.3389/fneur.2022.685636

**Published:** 2022-05-16

**Authors:** Szu-Ying Lin, Po-Chen Lin, Yi-Cheng Lin, Yi-Jung Lee, Chen-Yu Wang, Shih-Wei Peng, Pei-Ning Wang

**Affiliations:** ^1^Department of Neurology, Taipei Municipal Gan-Dau Hospital, Taipei, Taiwan; ^2^Doctoral Degree Program of Translational Medicine, National Yang Ming Chiao Tung University and Academia Sinica, Hsinchu, Taiwan; ^3^Department of Neurology, Neurological Institute, Taipei Veterans General Hospital, Taipei, Taiwan; ^4^Institute of Neuroscience, School of Life Sciences, National Yang-Ming Chiao Tung University, Taipei, Taiwan; ^5^Division of Neurology, Department of Medicine, Taipei City Hospital Renai Branch, Taipei, Taiwan; ^6^Institute of Brain Science, National Yang-Ming Chia Tung University, Taipei, Taiwan; ^7^Division of General Neurology, Department of Neurology, Neurological Institute, Taipei Veterans General Hospital, Taipei, Taiwan; ^8^Brain Research Center, National Yang-Ming Chia Tung University, Taipei, Taiwan; ^9^Aging and Health Research Center, National Yang-Ming Chia Tung University, Taipei, Taiwan; ^10^Department of Neurology, School of Medicine, National Yang-Ming Chia Tung University, Taipei, Taiwan

**Keywords:** early mild cognitive impairment (EMCI), mild cognitive impairment (MCI), Alzheimer's Disease, longitudinal follow-up, cognitive tests

## Abstract

**Introduction:**

Amnestic mild cognitive impairment (MCI) can be classified as either early MCI (EMCI) or late MCI (LMCI) according to the severity of memory impairment. The aim of this study was to compare the prognosis and clinical course between EMCI and LMCI.

**Methods:**

Between January 2009 and December 2017, a total of 418 patients with MCI and 146 subjects with normal cognition were recruited from a memory clinic. All the patients received at least two series of neuropsychological evaluations each year and were categorized as either EMCI or LMCI according to Alzheimer's Disease Neuroimaging Initiative 2 (ADNI2) criteria.

**Results:**

In total, our study included 161 patients with EMCI, 258 with LMCI, and 146 subjects with normal cognition as controls (NCs). The mean follow-up duration was 3.55 ± 2.18 years (range: 1–9). In a first-year follow-up assessment, 54 cases (32.8%) of EMCI and 16 (5%) of LMCI showed a normal cognitive status. There was no significant difference between the first year EMCI reverter and NCs in terms of dementia-free survival and further cognitive decline. However, first-year LMCI reverters still had a higher risk of cognitive decline during the following evaluations. Until the last follow-up, annual dementia conversion rates were 1.74, 4.33, and 18.6% in the NC, EMCI, and LMCI groups, respectively. The EMCI and LMCI groups showed a higher rate of progression to dementia (log-rank test, *p* < 0.001) than normal subjects. Compared with NCs, patients in the LMCI group showed a significantly faster annual decline in global cognition [annual rate of change for the mini-mental status examination (MMSE) score: −1.035, *p* < 0.001]) and all cognitive domains, while those in the EMCI group showed a faster rate of decline in global cognitive function (annual rate of change for the MMSE score: −0.299, *p* = 0.001).

**Conclusion:**

It is important to arrange follow-up visits for patients with MCI, even in the EMCI stage. One-year short-term follow-up may provide clues about the progression of cognitive function and help to identify relatively low-risk EMCI subjects.

## Introduction

Mild cognitive impairment (MCI) is a state of cognition that occurs between normal aging and dementia (1). Because Alzheimer's Disease (AD) and several other types of neurodegenerative disease have a long preclinical phase involving the progressive accumulation of pathological changes in the brain, MCI can be considered the earliest detectable symptom of dementia (2, 3).

The assessment of MCI was initially aimed at detecting the risk of AD in patients for research purposes. MCI is defined as a decline in cognitive abilities with objective evidence of impairment in standard memory or other cognitive tests but without the significant impairment in daily activities seen in conditions such as dementia (1). Impaired performance on cognitive testing is usually defined as an episodic memory performance score that falls below 1.5 *SD* of the age and education-adjusted norm. To further understand the clinical course of MCI, the Alzheimer's Disease Neuroimaging Initiative (ADNI) (4) began to define an earlier stage of MCI in 2009, which was referred to as early MCI (EMCI) (4). EMCI refers to a cognitive impairment that falls between 1 and 1.5 *SD* below the normative mean on a standard test (4). Except in cases of minor cognitive changes, EMCI has been associated with amyloid deposition and brain metabolism (4), functional network breakdown (5), and brain volume changes (6). However, longitudinal outcomes are still under debate.

The ADNI reported that there was a higher risk of AD-associated dementia in both LMCI and EMCI subjects, with an annual conversion rate from MCI to dementia of 17.5% for LMCI and 2.3% for patients with EMCI (7). The AgeCoDe study (a German study on aging, cognition, and dementia in patients in primary care) also reported an increased risk of AD that was highest in patients with LMCI [hazard ratio (HR) = 7.27; *p* < 0.001], followed by patients with EMCI (HR = 3.1, *p* < 0.001), and those with subjective memory impairment (HR = 1.55, *p* = 0.04) (8). Compared with patients with LMCI, those with EMCI exhibited more heterogeneous characteristics and a higher likelihood of displaying negative indicators of AD pathology (e.g., lower progression rates and cerebrospinal fluid (CSF) biomarkers) (9).

Subjects with MCI exhibit a faster decline in various cognitive domains when compared with those undergoing the normal aging process. One study showed that the rate of cognitive decline began to accelerate approximately 5 to 6 years before dementia diagnosis and increased modestly from approximately 4 to 6 years before the confirmation of MCI (10). Patients with LMCI are known to experience a faster decline and linear change in terms of episodic memory, semantic memory, and perceptual speed (11). In contrast, delayed recall, working memory, and spatial memory all showed a rapid decline before the onset of dementia (12). Other studies reported that impaired episodic memory in patients with LMCI was associated with an increased risk of AD and a faster cognitive decline (13).

Compared with the well-known LMCI disease model, relatively few studies have focused on longitudinal changes and changes in specific cognitive domains. Studies have shown that during the EMCI stage, baseline cognitive function and amyloid- and APOE ε4-positive status were correlated with poor cognitive and functional outcomes (14). However, very little is known about the differences in cognitive domain-specific alterations between normal controls and subjects with either EMCI or LMCI.

In this study, we investigated the natural history and prognosis of patients with EMCI. We further compared the characteristics and annual changes within each cognitive domain among the EMCI, LMCI, and normal cognition (NC) groups of subjects.

## Materials and Methods

This study was conducted at the Taipei Veterans General Hospital between January 2009 and December 2017. The study was approved by the Local Ethics Committee of Human Research in Taipei Veterans General Hospital, Taiwan. Each participant received standardized clinical, neurological, and neuropsychological examinations. Informed written consent was obtained from all participants.

### Participants

Subjects were recruited from neurological clinics. Experienced neurologists interviewed all the participants. The diagnosis of early or late MCI was based on data obtained through clinical interviews, neurological examinations, neuropsychological tests, laboratory findings, and neuroimaging evaluation. The Geriatric Depression Scale (GDS) (short form) was done at the first visit to exclude geriatric depression (15). Laboratory and MRI examinations were used to exclude, but not diagnose, other major neuropathologies, including tumors, strokes, severe white matter disease, or inflammation. None of the subjects had a history of major brain trauma, brain tumor, stroke, epilepsy, alcoholism, major psychiatric illness, or other systemic diseases that could affect cognitive function. The NC subjects were all volunteers and were free of neurological disease or any form of cognitive complaint. All the participants received annual neuropsychological examinations (mean duration: 12 ± 3 months) until they progressed to dementia. An experienced neurologist diagnosed each subject upon follow-up based on changes in the logical memory score and the daily function impairment for dementia. Subjects were excluded if they did not attend the follow-up evaluation.

### Clinical Assessments

A trained neuropsychologist conducted a series of neuropsychological assessments on an annual basis.

Global cognitive function was evaluated by the mini-mental state examination (MMSE) (16) while episodic memory was assessed by the Chinese version of the Wechsler Memory Scale–Logical Memory subtest (WMS-LM) (17). Verbal memory was tested by the Chinese version of the Verbal Learning Test (CVVLT; featuring 9 items, 4 trials, and a 10-min delayed recall test) (18). Visual memory was investigated by determining scores for a 10-min recall test in the modified Rey-Osterrieth Complex Figure Test (CFT) (19). Language function was assessed by the Chinese version of the 30-item Boston Naming Test (BNT-30) (20) and a categorical verbal fluency (VF) test that involved naming as many animals as possible within 1 min. The forward digit span was used to evaluate attention. Executive function was investigated using the backward digit span and the modified Trail Making Test parts A and B (TMT-A and TMT-B; the subjects had a maximum of 120 s to complete the examination) (21).

### Diagnosis of EMCI, LMCI, and MCI Reversion

The diagnosis of EMCI and LMCI was based on the criteria proposed by Petersen et al. (22) including (1) the presence of memory complaints that were preferably corroborated by an informant; (2) impaired episodic memory function as documented by the Chinese Version of the WMS-LM (delayed paragraph recall). The EMCI group was defined using a cutoff score of 5–8 (1–1.5 *SD*s from normative values), while the LMCI group was determined by scores below 5 (<1.5 *SD*s from normative data); (3) the ability of the patient to maintain daily living activities, including social and familial activities, according to clinical judgment; (4) the preservation of general cognitive function, as determined by both clinical impression and an MMSE score above a cut-off value of 24, the reference limit in Taiwan (23). Only patients with a clinical dementia rating (CDR) score of 0.5 or less and a score of 0.5 on the memory domain were recruited.

Subjects with MCI reversion were defined as those who were initially diagnosed with EMCI or LMCI but subsequently regained normal memory function (with normal episodic memory, a WMS-LM score > 9 grades, and normal daily function) in their second year (the subjects' first return visit to the clinic 9 to 15 months after recruitment).

### Clinical Data Analysis

Data analysis was carried out using the SPSS software, version 26 for Mac (SPSS Inc., Chicago, IL, USA). A two-tailed *p*-value < 0.05 was considered to be statistically significant. Descriptive statistics derived from demographic data were presented as means ± *SD*. ANOVA was used to analyze the numerical data such as age and educational level. A *post-hoc* comparison was done using the Fisher's Least Significant Difference (LSD) test. The chi-squared test and the Fisher's exact test were used to analyze the categorical data. The scores of neuropsychological tests between groups were compared by the analysis of covariance (ANCOVA) using age, gender, and education as the covariates. The probability of remaining dementia-free during the follow-up periods was determined using the Kaplan–Meier survival curves using log-rank statistics.

The repeated measurement data for each neuropsychological test were assessed using the generalized estimating equations (GEE) model (17). To allow for the analysis of multiple visits by the same subjects and to correct for changing values for a single individual over time, the follow-up period (in years) was used as a covariate, controlling for age and educational level. First, mean changes over time were compared between patients with MCI and normal controls. The linear slope was equal to the annual progression rate for the score in each neuropsychiatric test. Second, the interaction between time and each group (time × group) was assessed using the NC group as the reference. Beta regression coefficients and 95% CIs were determined using the robust covariance estimation; the statistical significance of these coefficients was tested by the Wald test.

## Results

In total, 564 participants were enrolled during the study period, including 146 normal controls, 161 subjects with EMCI, and 258 subjects with LMCI. In the secondary evaluation, 129 (88.3%) subjects with NC, 155 (96.2%) with EMCI, and 236 (91.47%) with LMCI remained eligible and were included in subsequent analysis. Seventy-eight (53.4%) NC, 91 (56.5%) EMCI, and 131 (50.7%) LMCI subjects completed more than 3 times annual follow-up. The mean follow-up period was 3.55 ± 2.18 years (range: 1–9.16).

Table 1 shows the demographic and baseline neuropsychological characteristics of the study participants. The three groups were similar with regard to gender. The MCI subjects were significantly older (NC: 68.6 ± 8.11 years, EMCI: 73.7 ± 8.39 years, LMCI: 75.2 ± 7.66 years; *p* < 0.001) and had a significantly lower level of education (NC: 13.1 ± 3.61 years, EMCI: 11.8 ± 3.87 years, LMCI: 10.5 ± 4.63 years; *p* < 0.001) than the NC subjects. The effects of age and education were controlled for by ANCOVA in all the subsequent analyses involving comparisons between the baseline and the changing values of the neuropsychological tests.

**Table 1:** Demographic data and baseline neuropsychological performance.

	**Normal (*n* = 146)**	**Early MCI (*n* = 161)**	**Late MCI (*n* = 258)**	***P*-value**
Age (years)	68.6 ± 8.11	73.7 ± 8.39	75.2 ± 7.66	<0.001^a, b, c^
Education (years)	13.1 ± 3.61	11.8 ± 3.87	10.5 ± 4.63	<0.001^a, b, c^
Gender (male, %)	71 (40.1)	93 (50.0)	127 (46.0)	0.164
MCI reverters (%)		54 (33.5)	17 (6.6)	
Follow-up (years)	3.63 ± 2.27	3.71 ± 2.21	3.42 ± 2.12	0.380
GDS	3.46 ± 3.54	4.12 ± 3.46	4.04 ± 3.21	0.162
MMSE	28.7 ± 1.29	27.69 ± 1.45	26.55 ± 1.60	<0.001^a, b, c^
STM	2.60 ± 0.63	2.04 ± 0.93	1.54 ± 1.02	<0.001^a, b, c^
WMS-LM	12.87 ± 2.96	7.20 ± 1.45	2.25 ± 1.78	<0.001^a, b, c^
CVVLT total recall	28.34 ± 4.11	24.67 ± 4.86	21.02 ± 4.60	<0.001^a, b, c^
CVVLT delay recall	7.64 ± 1.50	6.12 ± 2.13	3.92 ± 2.48	<0.001^a, b, c^
CFT immediate recall	20.36 ± 6.75	14.97 ± 7.02	9.32 ± 6.53	<0.001^a, b, c^
CFT delay recall	19.56 ± 7.35	15.14 ± 6.95	8.56 ± 6.58	<0.001^a, b, c^
CFT copy	32.14 ± 3.11	30.74 ± 3.84	29.40 ± 5.19	0.269
Clock drawing	9.77 ± 0.59	9.52 ± 0.94	9.49 ± 0.92	0.504
BNT	28.40 ± 2.11	27.13 ± 2.98	25.81 ± 3.47	<0.001^a, b, c^
Forward digit scan	8.38 ± 0.81	8.18 ± 0.99	7.98 ± 1.10	0.001
Backward digit scan	5.29 ± 1.37	4.59 ± 1.35	4.21 ± 1.17	<0.001^a, b^
VF-animal naming	17.61 ± 4.64	15.02 ± 4.56	13.52 ± 4.07	<0.001^a, b, c^
TMT-A (s)	14.99 ± 7.67	19.71 ± 10.56	27.80 ± 18.46	<0.001^b, c^
TMT-A (line)	7 ± 0	7 ± 0	6.96 ± 0.40	0.712
TMT-B (s)	44.26 ± 26.8	66.03 ± 34.7	81.78 ± 34.8	<0.001^a, b, c^
TMT-B (line)	13.67 ± 1.67	12.95 ± 2.68	11.34 ± 4.26	0.001^b, c^

*Values are expressed as means ± SD. ANOVA with a least significant difference post-hoc test was used for demographic data, ANCOVA with a least significant difference post-hoc test was used for neuropsychological performance. p < 0.05*;

a *normal vs. EMCI*;

b *normal vs. LMCI*;

c *EMCI vs. LMCI*.

*NC, normal cognition; MCI, early mild cognitive impairment; GDS, Geriatric Depression Scale; MMSE, mini-mental status examination; STM, short-term memory; WMS-LM, Wechsler memory scale-logical memory; CVVLT, Chinese version of the verbal learning test; CFT, complex figure test; BNT, Boston naming test; VF, verbal fluency; TMT, trail making test*.

### The Outcomes for the NC, EMCI, and LMCI Groups

**Figure 1:**
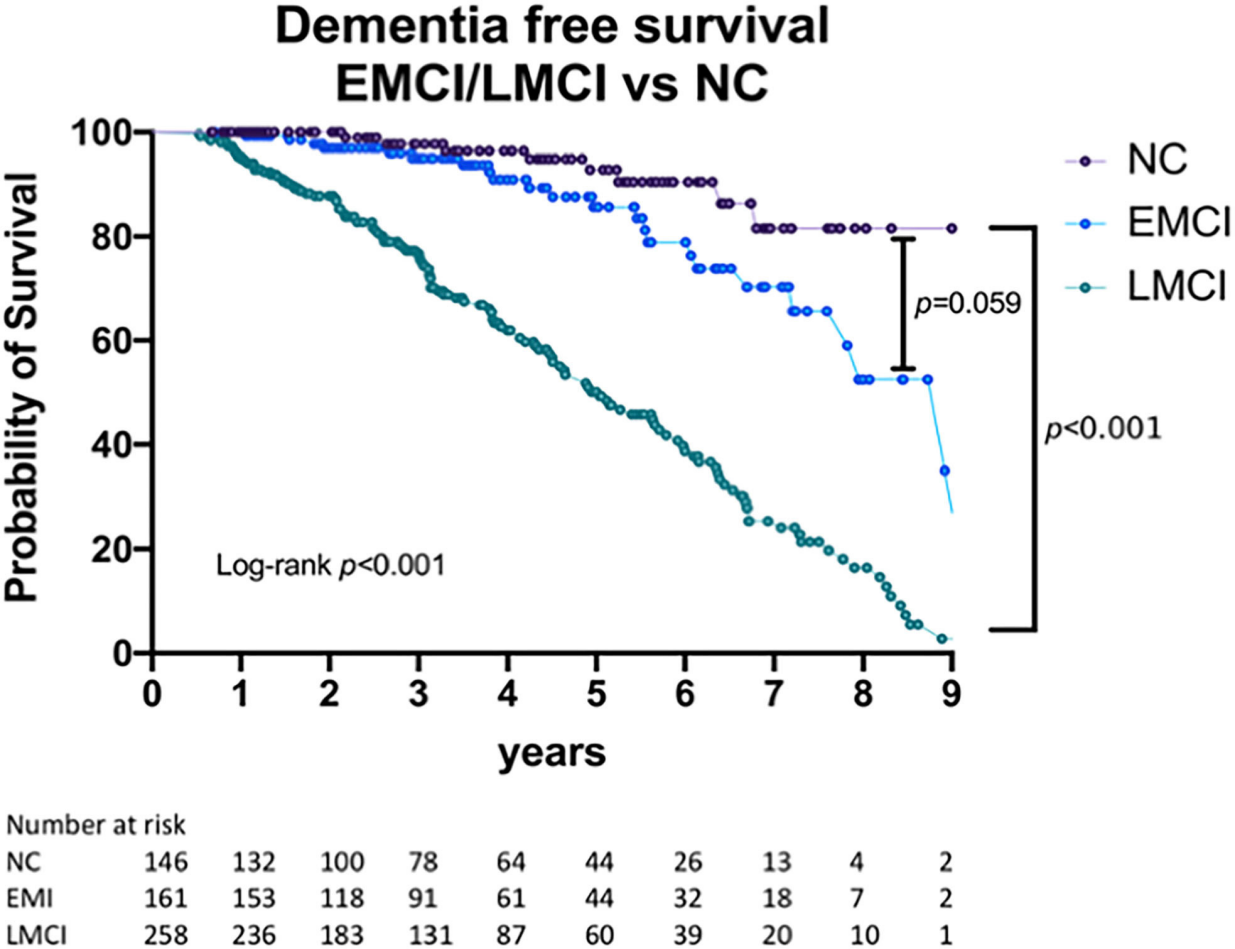
Dementia-free survival for NC, EMCI, and LMCI participants. NC, normal cognition; EMCI, early mild cognitive impairment; LMCI, late mild cognitive impairment.

In the second year after recruitment (at the time of the first follow-up), 61 (39.3%) subjects with EMCI and 16 (5%) with LMCI had shown reversal to a normal cognitive status. In longer follow-up, 16 EMCI subjects underwent reversion at the third-year follow-up (4 subjects converted to EMCI status in the next follow-up, others remained with normal cognitive status during the follow-up periods). One LMCI subject underwent reversion at the third-year' follow-up. At the time of the last evaluation in 2018, nine (6.16%) of the subjects with NC, 24 (14.9%) of the subjects with EMCI, and 124 (48%) of the subjects with LMCI had progressed to dementia. The annual conversion rate to dementia was 1.74% in the NC group, 4.33% in the EMCI group, and 18.6% in the LMCI group. Of these, 7 (4.79%) subjects with NC, 16 (9.9%) with EMCI, and 104 (40.3%) with LMCI had developed Alzheimer's dementia. Table 2 shows a detailed breakdown of the dementia subtypes at the end of the follow-up period, while Figure 1 shows the dementia-free survival for the three groups. Compared with the other two groups, subjects with LMCI were associated with a significantly shorter dementia-free survival time (log-rank test, *p* < 0.001). The median dementia-free survival times for EMCI and LMCI subjects were 8.917 (95% CI = 7.505–10.329) and 5.043 (95% CI = 4.236–5.850) years, respectively. Compared with those in the NC group, subjects with LMCI had a significantly higher risk of dementia (HR = 8.812; 95% CI = 4.477–17.344, *p* < 0.001). We also identified a trend for higher risk of dementia in EMCI participants (HR = 2.109; 95% CI = 0.971–4.592, *p* = 0.059), although this trend was not statistically significant.

**Table 2:** Dementia outcome and clinical subtype.

**Subgroup**	**NC**	**EMCI**	**LMCI**	***P*-value**
Subjects diagnosed with dementia ultimately	9	24	124	
AD	7 (77.8%)	16 (66.7%)	104 (83.9%)	0.142
Non-AD dementia	2 (22.2%) 1 VD, 1 FTD	8 (33.3%) 3 VD, 3 DLB 1 PDD, 1 FTD	20 (16.1%) 8 VD, 5 DLB 3 PDD, 4 FTD	

*The chi-squared test was used to analyze categorical data. AD, Alzheimer's Disease; DLB, dementia with Lewy bodies; EMCI, early mild cognitive impairment; FTD, frontal temporal dementia; LMCI, late mild cognitive impairment; NC, normal control; PDD, Parkinson's disease with dementia; VD, vascular dementia*.

### The Outcomes for the Subjects With EMCI and LMCI Undergoing Reversion

**Figure 2:**
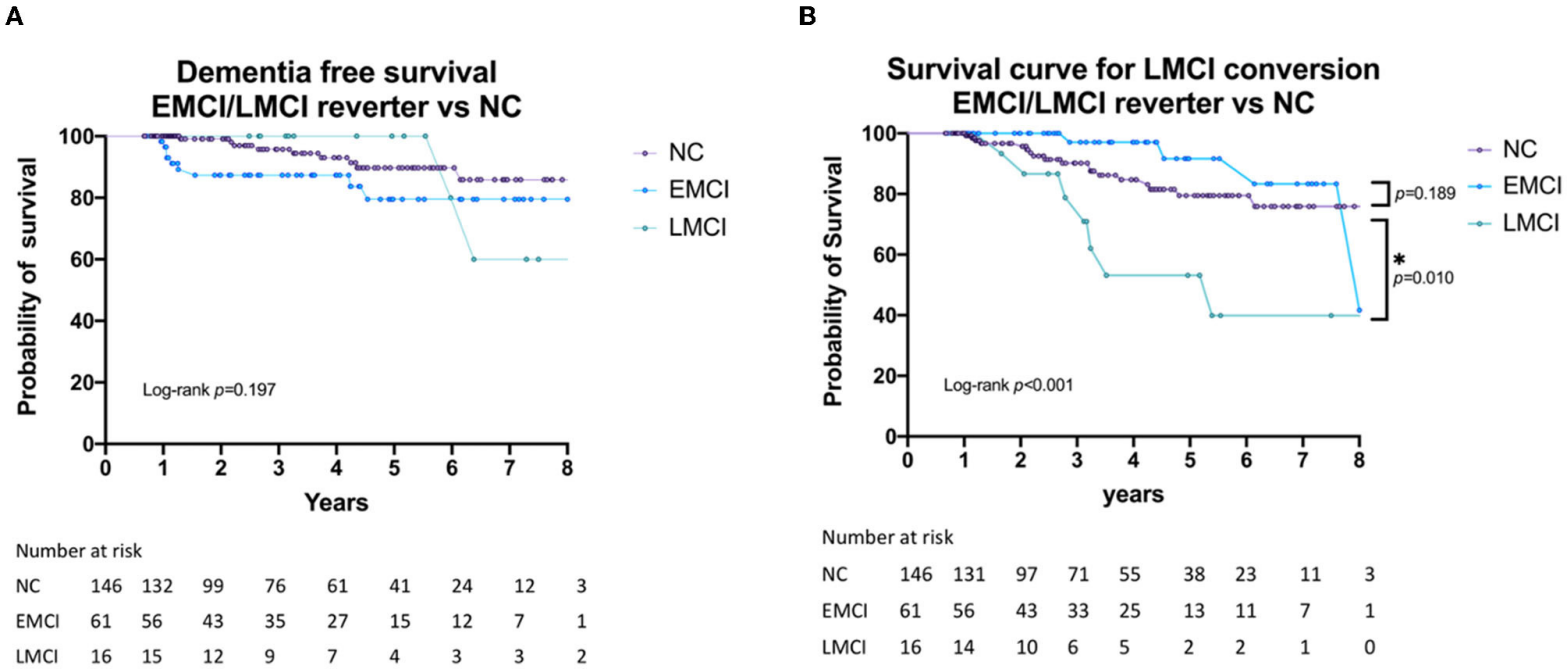
**(A)** Dementia-free survival of EMCI/LMCI who underwent reversion to normal cognitive status compared with that for normal controls. **(B)** LMCI-free survival of EMCI/LMCI who underwent revision to normal cognitive status compared with that for the NC group. NC, normal cognition; EMCI, early mild cognitive impairment; LMCI, late mild cognitive impairment. *: *p* < 0.05.

Figure 2A shows the dementia-free survival times for the subjects with EMCI or LMCI who underwent reversion as compared with that of the NC group. Figure 2B shows the survival curves of the time LMCI, who underwent reversion to normal cognitive status, converted to LMCI again when compared with the time taken for normal controls to develop LMCI. Subjects with LMCI who underwent reversion exhibited a significantly poorer outcome during the follow-up period (log-rank, *p* < 0.001 for secondary LMCI conversion). Although not significant, the EMCI subjects who underwent reversion showed a trend for a better prognosis compared with those of the NC group.

### Annual Changes in Cognitive Task Scoring in NC, EMCI, and LMCI Subjects

**Figure 3:**
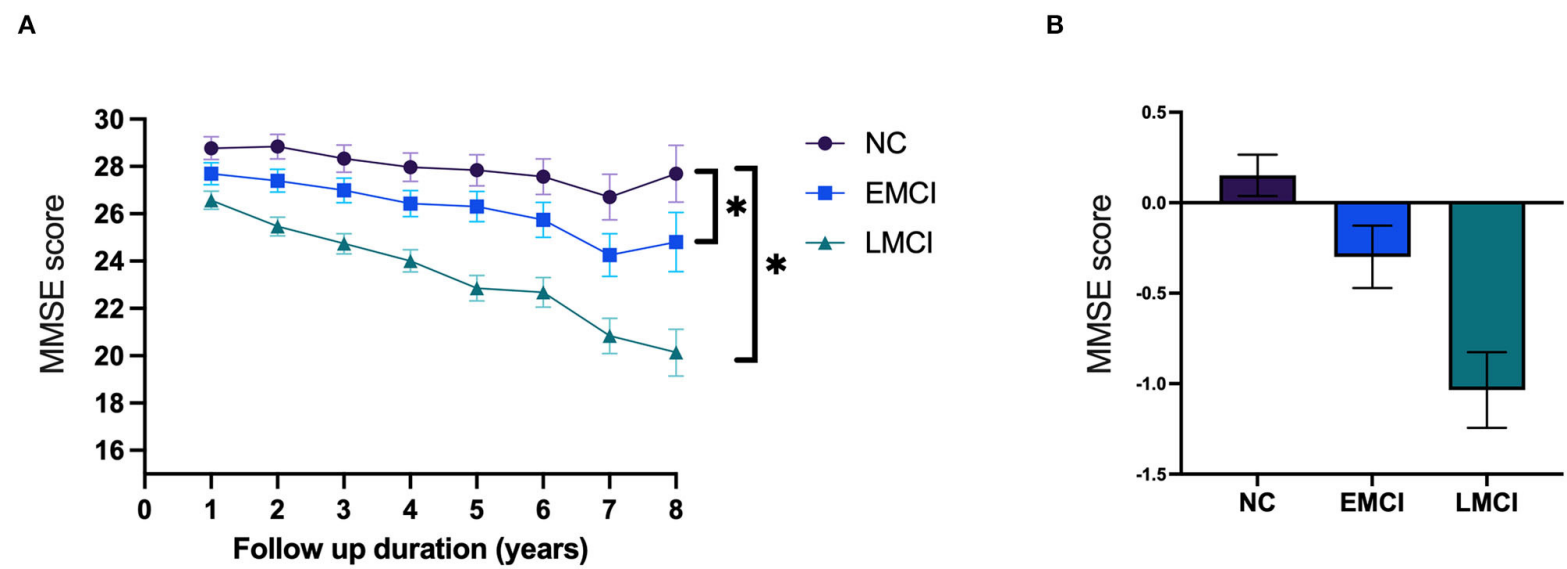
**(A)** The prediction of annual changes for the mini-mental status examination (MMSE) score. **(B)** The annual rate of change for MMSE. NC, normal cognition; EMCI, early mild cognitive impairment; LMCI, late mild cognitive impairment. *: *p* < 0.05.

Table 3 shows the annual change for each cognitive task in the three groups. The annual rates of change for the MMSE score were −1.035 (*p* < 0.001) for the LMCI subjects and −0.299 (*p* = *0.0*01) for the EMCI subjects (Figure 3). The annual rates of change for the WMS-LM score were −2.13 (*p* < 0.001) for the LMCI subjects and −1.086 (*p* < 0.001) for the EMCI subjects. The LMCI group showed a significant decline in all neuropsychological test scores over time except for the forward digit span. Meanwhile, subjects in the EMCI group displayed a significant annual decline in global cognitive function (MMSE: −0.299, *p* = 0.001), memory [short-term memory (STM): −0.101, *p* < 0.001, WMS-LM: −1.086, *p* < 0.001, CVVLT total recall: −0.51, *p* = 0.003, CVVLT delayed recall: −0.307, *p* < 0.001], visual memory (CFT delayed recall: −0.91, *p* = 0.002), and the more difficult executive function tests (TMT-B: 2.381, *p* = 0.007, visual fluency: −0.421, *p* = 0.003, but not in TMT-A:0.132, *p* = 0.713). The annual rate of change for each neuropsychological test is shown in Supplementary Table 1 and Figure 1. As shown in Table 3, when compared with those of the NC group, subjects with LMCI exhibited a significantly greater annual rate of change in the MMSE score (*p* < 0.001), STM (*p* = 0.003), CVVLT total recall (*p* = 0.008), CFT immediate recall (*p* = 0.043), clock-drawing ability (*p* = 0.013), and VF (*p* = 0.034). Subjects in the EMCI group exhibited a significantly faster rate of decline for global function (MMSE, *p* = 0.047) but not for the other neuropsychological test parameters. Compared with EMCI subjects, LMCI subjects had a trend of faster decline in all neuropsychological tests except for digital forward (Supplementary Figure 1, Supplementary Table 2).

**Table 3:** Generalized Estimating Equations (GEE) analysis of the annual change in each neuropsychological test.

**Variable**	**Regression coefficient**	**SE**	**95% CI**	**χ^2^**	***P*-value**
**MMSE**
Diagnosis*time (LMCI *vs*. NC)	−0.653	0.106	−0.861~−0.445	37.883	<0.001
Diagnosis*time (EMCI *vs*. NC)	−0.184	0.0927	−0.366~−0.003	3.969	0.047
**STM**
Diagnosis*time (LMCI *vs*. NC)	−0.077	0.0261	−0.128~−0.026	8.699	0.003
Diagnosis*time (EMCI *vs*. NC)	−0.025	0.0302	−0.084~0.035	0.662	0.416
**WMS Logical memory**
Diagnosis*time (LMCI *vs*. NC)	−0.117	0.1635	−0.438~0.204	0.511	0.475
Diagnosis*time (EMCI *vs*. NC)	−0.249	0.1998	−0.641~0.142	1.557	0.212
**CVVLT total recall**
Diagnosis*time (LMCI *vs*. NC)	−0.448	0.1696	−0.781~−0.116	6.992	0.008
Diagnosis*time (EMCI *vs*. NC)	−0.056	0.1929	−0.434~0.322	0.084	0.771
**CVVLT delayed recall**
Diagnosis*time (LMCI *vs*. NC)	−0.127	0.0833	−0.290~0.036	2.331	0.127
Diagnosis*time (EMCI *vs*. NC)	−0.086	0.0852	−0.253~0.081	1.027	0.311
**CFT immediate recall**
Diagnosis*time (LMCI *vs*. NC)	−0.589	0.2911	−1.159~−0.018	4.088	0.043
Diagnosis*time (EMCI *vs*. NC)	−0.431	0.3073	−1.034~0.171	1.971	0.16
**CFT delayed recall**
Diagnosis*time (LMCI *vs*. NC)	−0.562	0.2967	−1.144~0.019	3.593	0.058
Diagnosis*time (EMCI *vs*. NC)	−0.463	0.3206	−1.091~0.165	2.085	0.149
**CFT copy**
Diagnosis*time (LMCI *vs*. NC)	−0.158	0.1295	−0.412~0.095	1.497	0.221
Diagnosis*time (EMCI *vs*. NC)	−0.038	0.1095	−0.253~0.177	0.120	0.729
**Clock drawing**
Diagnosis*time (LMCI *vs*. NC)	−0.062	0.025	−0.111~−0.013	6.109	0.013
Diagnosis*time (EMCI *vs*. NC)	−0.005	0.018	−0.040~0.031	0.067	0.796
**BNT**
Diagnosis*time (LMCI *vs*. NC)	−0.12	0.1003	−0.317~0.076	1.437	0.231
Diagnosis*time (EMCI *vs*. NC)	−0.054	0.0781	−0.208~0.099	0.485	0.486
**Forward digit scan**
Diagnosis*time (LMCI *vs*. NC)	0.016	0.0287	−0.040~0.072	0.305	0.581
Diagnosis*time (EMCI *vs*. NC)	−0.026	0.0345	−0.094~0.041	0.586	0.444
**Backward digit scan**
Diagnosis*time (LMCI *vs*. NC)	0.012	0.0355	−0.054~0.077	0.125	0.724
Diagnosis*time (EMCI *vs*. NC)	0.035	0.0348	−0.033~0.103	1.005	0.316
**TMT-A (s)**
Diagnosis*time (LMCI *vs*. NC)	0.464	0.4141	−0.348~1.275	1.253	0.263
Diagnosis*time (EMCI *vs*. NC)	−0.031	0.3583	−0.733~0.671	0.007	0.931
**TMT-B (s)**
Diagnosis*time (LMCI *vs*. NC)	1.398	0.8834	−0.334~3.129	2.503	0.114
Diagnosis*time (EMCI *vs*. NC)	0.759	0.883	−0.971~2.490	0.739	0.39
**Verbal fluency**
Diagnosis*time (LMCI *vs*. NC)	−0.353	0.1664	−0.679~−0.027	4.492	0.034
Diagnosis*time (EMCI *vs*. NC)	−0.09	0.1648	−0.413~0.233	0.300	0.584

*NC, normal cognition; EMCI, early mild cognitive impairment; LMCI, late mild cognitive impairment; GDS, Geriatric Depression Scale; MMSE, mini-mental status examination; STM, short-term memory; WMS-LM, Wechsler memory scale-logical memory; CVVLT, Chinese version of the verbal learning test; CFT, complex figure test; BNT, Boston naming test; TMT, trail making test*.

## Discussion

Although the structural and functional changes during the early stages of AD have been extensively studied, little is known about the detailed changes in clinical outcomes for subjects with EMCI. Here, we demonstrated that both LMCI and EMCI subjects have a higher risk of dementia and global functional decline over follow-up periods lasting up to 8 years when compared with NC subjects. Patients with LMCI who underwent reversion to a normal cognitive status showed poor cognitive outcomes, in contrast to that seen in the EMCI reverter, suggesting that the risk of long-term cognitive decline is increased once LMCI is diagnosed. Meanwhile, a 1-year short-term follow-up may help to identify relatively low-risk EMCI subjects. Our results indicated that AD comprises a clinical spectrum that initially features memory impairment, and that the risks associated with this disease increase as the disease progresses.

Compared with that in the NC group, the risk of dementia was significantly increased in the LMCI group (HR = 8.812, *p* < 0.001) and showed an increasing trend in EMCI subjects (HR = 2.109, *p* = 0.059). At the last follow-up, 14.9% of the EMCI subjects had converted to dementia, with a median dementia-free survival period of 8.9 years. The annual conversion rate to dementia was 1.74% in the NC group, 4.33% in the EMCI group, and 18.6% in the LMCI group. The conversion rate for the EMCI group was slightly higher than that reported by the ADNI (2.3%) (7). Notably, in our study, the conversion rate to dementia included all types of dementia subtypes and not just AD.

The EMCI subjects, who exhibit minor episodic memory impairment, showed high levels of heterogeneity in the underlying pathological changes. AD dementia occurred in 66% of subjects in the EMCI group and 83% of subjects in the LMCI group. Studies have reported a faster decline in subjects with a greater degree of AD pathology, including increased APOE ε4 and amyloid positivity (24). It is a possible higher rate of other neurodegenerative pathologies rather than AD in the wider standard (9).

Compared with those in the NC group, LMCI subjects showed a faster decline in global cognitive function (MMSE), memory (STM, CVVLT total recall), visuospatial memory (clock drawing test, CFT immediate recall), and executive function (VF test). Several studies (25–27) have reported a mean annual rate of decline for the MMSE score of 1.8–6.7 for probable AD, which is slower in less severe dementia. The MMSE score decreased by 1 point each year (−1.035, *p* < 0.001) in the LMCI group and by a lower amount in the EMCI group (−0.299, *p* = 0.001). Episodic memory deficit is often the first manifestation of AD. Both the verbal learning test and the WMS-LM test can be used to predict a further decline (28, 29). In this study, although the baseline episodic memory (WMS-LM and CVVLT delayed recall) scores in the LMCI group were low, they still showed a significant annual decline (WMS-LM: −2.13 per year, *p* < 0.001; CVVLT delayed recall: −0.928, *p* < 0.001).

The MCI subjects showed a significant decline in more difficult executive tasks, including the TMT-B (5.844, *p* < 0.001 for the LMCI group; 2.381, *p* = 0.007 for the EMCI group) and VF tasks (−1.044, *p* < 0.001 for the LMCI group; −0.421, *p* = 0.003 for the EMCI group). The aging brain is associated with a reduced prefrontal lobe volume and reduced levels of brain connectivity (30), changes that mainly manifest as executive dysfunction such as the loss of perceptual speed as assessed by the TMT (31). MCI subjects (defined as LMCI subjects in this study) have also been reported to undergo a faster decline in executive function compared with normal controls (32). The VF task can detect mild cases of AD (33). Patients with MCI presenting with a phonemic advantage were also reported to exhibit a higher risk of progression to AD (34). Apart from inhibition ability, a component of executive control, the VF task also measures language domains, including vocabulary size and lexical access speed (35). The VF task is an executive function task including language and other cognitive components that might be more sensitive for the detection of degenerative conditions in MCI subjects.

Neuropsychological tests and different MCI subtypes are associated with a high predictive value for dementia conversion, with sensitivities that range from 80.8 to 100% depending on the dementia type (2, 36–38). Over the long term, almost half of the MCI participants stabilized or reverted to normal cognition (39, 40). Baseline biomarkers that are indicative of amyloid deposition in the brain and neurodegeneration have been shown to predict dementia conversion in patients with amnestic MCI (41) and produce a low number of false positives (42). However, high costs and extensive technical requirements create significant limitations for functional brain imaging or CSF exams for research. Blood-based amyloid biomarkers represent an alternative with higher levels of accessibility and a high diagnostic value (43–45), and standardizing and validating additional blood-based biomarkers is currently the focus of intensive research (45). During longitudinal follow-up, a decline in executive function during the preclinical stage and a deterioration in memory function during the MCI stage have been associated with decreased levels of complex instrumental activities of daily living (46). Repeat neuropsychological tests that are focused on verbal memory and semantic processing provide an affordable and widely accessible tool for screening and evaluation (47).

At the first-year follow-up, subjects with EMCI who underwent reversion presented with a similar prognosis for cognitive outcome and risk of dementia as the NC group. Ultimately, EMCI subjects showed a trend for higher risk of dementia conversion and global cognitive decline, although this risk was not statistically significant. The rate of cognitive change in subjects with EMCI was not significantly different when compared with that of normal controls. In contrast to EMCI reverters, the LMCI subjects who underwent reversion at first-year follow-up remained at increased risk of further cognitive decline. LMCI may be associated with the more severe underlying neurodegenerative process. Those neurodegenerative processes remained active even when the clinical symptoms presented transient improvement and would lead to future cognitive decline (40). Neuropsychological examinations carried out over short-term follow-ups might help identify patients with better prognoses. Other factors, such as resiliency, still require further investigation.

This study had some limitations. First, we recruited MCI subjects according to cognitive evaluations rather than disease-specific biomarkers. Without the above biomarkers, it is hard to confirm what proportion of the early and late MCI participants is on the AD trajectory. However, our MCI subjects met the criteria for the amnestic form of MCI, which is considered to be the prodromal form of AD. Subjects with obviously other neuropathologies were excluded by neurological examination, neuropsychological exam, or brain MRI image. Although our study included a heterogenous range of participants exhibiting different neurodegenerative pathologies, the major outcome was AD. The heterogeneity reflected clinical practice. Generally, the diagnosis of dementia arising from AD relies on a clinician's judgment. Most patients with dementia do not need to be confirmed by CSF analysis or the use of positron emission tomography (PET) amyloid biomarkers. Second, age and education level differed significantly among the different groups. The youngest subject and the highest education level were found in the NC group, while the oldest subject and the lowest educational level were found in the LMCI group. Consequently, we used ANCOVA for statistical analysis with the appropriate adjustments for age and educational level. Third, we used raw neuropsychological scores rather than *Z*-scores, which resulted in differences between each cognitive domain; however, the analysis of raw scores provided reference information for clinical follow-up. Fourth, ours was a hospital-based study, and this may have resulted in selection bias. Finally, there were small samples in both subgroups at longer follow-up. In this study, we conducted an annual neuropsychological evaluation for each NC, EMCI, and LMCI subgroup until the subjects converted to dementia. At 5-year follow-up, more than half of LMCI participants converted to dementia. Fewer sample sizes at longer than 4- to 5-year follow-up might be associated with shorter dementia-free survival in LMCI. A longer duration of follow-up may be required to correctly identify the differences between each of the study groups.

In conclusion, our analyses showed that LMCI and EMCI subjects have a higher risk of global cognitive decline and a trend for higher risk of dementia than normal controls. One-year, short-term follow-up might help to exclude and identify low-risk EMCI subjects. Besides, due to persistent cognitive decline, it is important to arrange a return visit, even during the EMCI stage. Longer follow-up is therefore needed to investigate the outcomes of subjects with LMCI and EMCI who show reversion to normal cognition.

## Data Availability Statement

The raw data supporting the conclusions of this article will be made available by the authors, without undue reservation.

## Ethics Statement

The studies involving human participants were reviewed and approved by Taipei Veteran General Hospital. The patients/participants provided their written informed consent to participate in this study.

## Author Contributions

PNW designed the study. SYL, PCL, YCL, and YJL acquired and analyzed the data. SYL and PNW wrote the manuscript. All authors contributed to the article and approved the submitted version.

## Funding

This research was financially supported by the Brain Research Center, National Yang-Ming Chia Tung University through The Featured Areas Research Center Program within the framework of the Higher Education Sprout Project by the Ministry of Education (MOE), Taiwan; the Ministry of Science and Technology, Taiwan (MOST 108-2321-B-010-013-MY2 and MOST 110-2321-B-010-007); and Taipei Veterans General Hospital (V108C-060 and V109C-027).

## Conflict of Interest

The authors declare that the research was conducted in the absence of any commercial or financial relationships that could be construed as a potential conflict of interest.

## Publisher's Note

All claims expressed in this article are solely those of the authors and do not necessarily represent those of their affiliated organizations, or those of the publisher, the editors and the reviewers. Any product that may be evaluated in this article, or claim that may be made by its manufacturer, is not guaranteed or endorsed by the publisher.

## Supplementary Material

Supplementary Figure 1 The prediction of annual changes for each neuropsychological test. **(A)** WMS-LM, **(B)** STM, **(C)** CVVLT total recall, **(D)** CVVLT delayed recall, **(E)** CFT copy, **(F)** CFT immediate recall, **(G)** CFT delayed recall, **(H)** BNT, **(I)** clock drawing, **(J)** forward digital span, **(K)** backward digit scan, **(L)** TMT-A, **(M)** TMT-B, and **(N)** VF. WMS-LM, Wechsler memory scale-logical memory; STM, short-term memory; CVVLT, Chinese version of the verbal learning test; CFT, complex figure test; BNT, Boston naming test; TMT, trail making test; VF, verbal fluency.

Supplementary Table 1 Generalized Estimating Equation (GEE) analysis of annual change rate in each neuropsychological test. NC, normal cognition; EMCI, early mild cognitive impairment; LMCI, late mild cognitive impairment; GDS, Geriatric Depression Scale; MMSE, mini-mental status examination; STM, short-term memory; WMS-LM, Wechsler memory scale-logical memory; CVVLT, Chinese version of the verbal learning test; CFT, complex figure test; BNT, Boston naming test; TMT, trail making test.

Supplementary Table 2 Generalized Estimating Equation (GEE) analysis of annual change rate differences between EMCI and LMCI in each neuropsychological test. NC, normal cognition; EMCI, early mild cognitive impairment; LMCI, late mild cognitive impairment; GDS, Geriatric Depression Scale; MMSE, mini-mental status examination; STM, short-term memory; WMS-LM, Wechsler memory scale-logical memory; CVVLT, Chinese version of the verbal learning test; CFT, complex figure test; BNT, Boston naming test; TMT, trail making test.
