# Supplementary figures and images for: The Clinical Course of Early and Late Mild Cognitive Impairment
Source: Front Neurol. 2022 May 16;13:685636. doi: 10.3389/fneur.2022.685636 (PMC9149311; doi:10.3389/fneur.2022.685636)

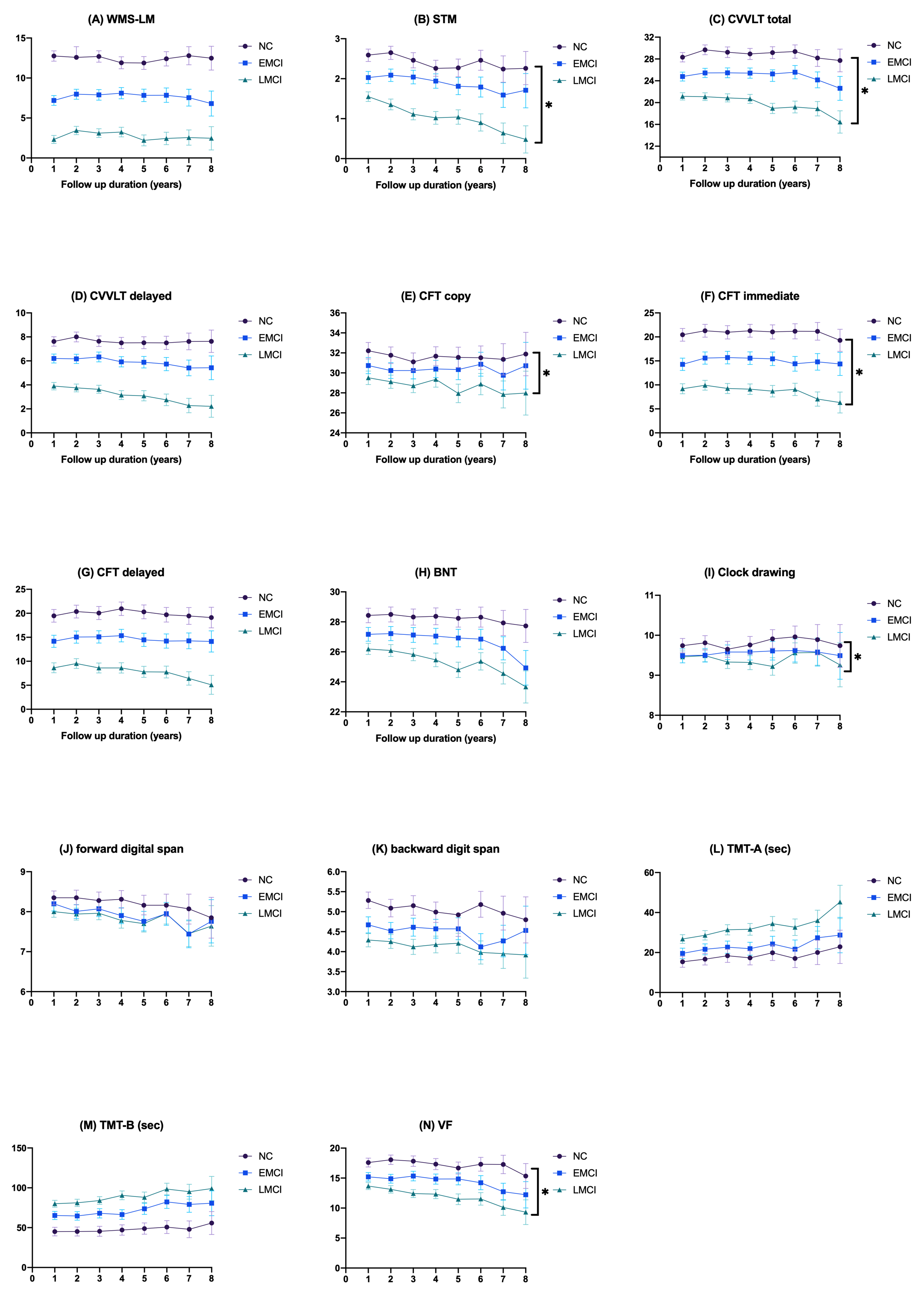

Supplement: Supplementary Figure 1 — The prediction of annual changes for each neuropsychological test. (A) WMS-LM, (B) STM, (C) CVVLT total recall, (D) CVVLT delayed recall, (E) CFT copy, (F) CFT immediate recall, (G) CFT delayed recall, (H) BNT, (I) clock drawing, (J) forward digital span, (K) backward digit scan, (L) TMT-A, (M) TMT-B, and (N) VF. WMS-LM, Wechsler memory scale-logical memory; STM, short-term memory; CVVLT, Chinese version of the verbal learning test; CFT, complex figure test; BNT, Boston naming test; TMT, trail making test; VF, verbal fluency. [file Image_1.TIFF]
