# Supplementary material for: The Clinical Course of Early and Late Mild Cognitive Impairment
Source: Front Neurol. 2022 May 16;13:685636. doi: 10.3389/fneur.2022.685636 (PMC9149311; doi:10.3389/fneur.2022.685636)
Supplement: Supplementary Table 1 — Generalized Estimating Equation (GEE) analysis of annual change rate in each neuropsychological test. NC, normal cognition; EMCI, early mild cognitive impairment; LMCI, late mild cognitive impairment; GDS, Geriatric Depression Scale; MMSE, mini-mental status examination; STM, short-term memory; WMS-LM, Wechsler memory scale-logical memory; CVVLT, Chinese version of the verbal learning test; CFT, complex figure test; BNT, Boston naming test; TMT, trail making test. [file Table_1.DOCX]

Supplementary: table 1 Generalized Estimating Equation (GEE) analysis of annual change rate in each neuropsychological test

| Variable | Regression coefficient | SE | 95% CI | χ 2 | p |
| --- | --- | --- | --- | --- | --- |
| MMSE |  |  |  |  |  |
| Intercept | 31.438 | 1.1186 | 29.246~33.631 | 789.863 | <0.001 |
| time | 0.151 | 0.0586 | 0.037~0.266 | 6.675 | 0.01 |
| Diagnosis*time (LMCI vs NC) | -1.035 | 0.1067 | -1.244~-0.826 | 94.074 | <0.001 |
| Diagnosis*time (EMCI vs NC) | -0.299 | 0.0877 | -0.471~-0.127 | 11.612 | 0.001 |
| STM |  |  |  |  |  |
| Intercept | 3.371 | 0.3441 | 2.697~4.046 | 95.992 | <0.001 |
| time | 0.105 | 0.0192 | 0.067~0.142 | 29.521 | <0.001 |
| Diagnosis*time (LMCI vs NC) | -0.297 | 0.0272 | -0.351~-0.244 | 119.038 | <0.001 |
| Diagnosis*time (EMCI vs NC) | -0.101 | 0.0284 | -0.156~-0.045 | 12.519 | <0.001 |
| WMS Logical memory |  |  |  |  |  |
| Intercept | 15.928 | 1.72 | 12.556~19.299 | 85.750 | <0.001 |
| time | 1.433 | 0.1406 | 1.158~1.709 | 103.952 | <0.001 |
| Diagnosis*time (LMCI vs NC) | -2.13 | 0.172 | -2.467~-1.793 | 153.384 | <0.001 |
| Diagnosis*time (EMCI vs NC) | -1.086 | 0.1894 | -1.458~-0.715 | 32.901 | <0.001 |
| CVVLT total recall |  |  |  |  |  |
| Intercept | 39.171 | 1.9678 | 35.314~43.027 | 396.253 | <0.001 |
| time | 1.263 | 0.1088 | 1.050~1.476 | 134.692 | <0.001 |
| Diagnosis*time (LMCI vs NC) | -1.793 | 0.175 | -2.136~-1.450 | 104.942 | <0.001 |
| Diagnosis*time (EMCI vs NC) | -0.51 | 0.1737 | -0.850~-0.170 | 8.621 | 0.003 |
| CVVLT delayed recall |  |  |  |  |  |
| Intercept | 10.008 | 1.0041 | 8.040~11.976 | 99.345 | <0.001 |
| time | 0.498 | 0.0454 | 0.409~0.587 | 120.157 | <0.001 |
| Diagnosis*time (LMCI vs NC) | -0.928 | 0.0881 | -1.100~-0.755 | 110.853 | <0.001 |
| Diagnosis*time (EMCI vs NC) | -0.307 | 0.0767 | -0.457~-0.157 | 16.004 | <0.001 |
| CFT immediate recall |  |  |  |  |  |
| Intercept | 31.067 | 3.3615 | 24.478~37.655 | 85.410 | <0.001 |
| time | 1.611 | 0.1741 | 1.270~1.952 | 85.678 | <0.001 |
| Diagnosis*time (LMCI vs NC) | -2.513 | 0.2839 | -3.069~-1.957 | 78.370 | <0.001 |
| Diagnosis*time (EMCI vs NC) | -1.028 | 0.2862 | -1.589~-0.467 | 12.907 | <0.001 |
| CFT delayed recall |  |  |  |  |  |
| Intercept | 31.699 | 3.3568 | 25.12-~38.278 | 89.175 | <0.001 |
| time | 1.517 | 0.1871 | 1.150~1.884 | 65.717 | <0.001 |
| Diagnosis*time (LMCI vs NC) | -2.455 | 0.2885 | -3.021~-1.890 | 72.452 | <0.001 |
| Diagnosis*time (EMCI vs NC) | -0.91 | 0.296 | -1.490~-0.330 | 9.456 | 0.002 |
| CFT copy |  |  |  |  |  |
| Intercept | 34.38 | 1.614 | 31.217~37.543 | 453.742 | <0.001 |
| time | 0.253 | 0.0685 | 0.119~0.387 | 13.661 | <0.001 |
| Diagnosis*time (LMCI vs NC) | -0.424 | 0.13 | -.678~-0.169 | 10.618 | 0.001 |
| Diagnosis*time (EMCI vs NC) | -0.127 | 0.1028 | -0.238~0.075 | 1.525 | 0.217 |
| Clock drawing |  |  |  |  |  |
| Intercept | 10.274 | 0.2913 | 9.703~10.845 | 1244.160 | <0.001 |
| time | 0.056 | 0.0101 | 0.036~0.075 | 30.320 | <0.001 |
| Diagnosis*time (LMCI vs NC) | -0.084 | 0.0277 | -0.022~0.006 | 11.702 | 0.002 |
| Diagnosis*time (EMCI vs NC) | -0.028 | 0.0152 | -0.058~0.002 | 3.375 | 0.066 |
| BNT |  |  |  |  |  |
| Intercept | 27.542 | 1.1281 | 25.331~29.754 | 596.067 | <0.001 |
| time | 0.278 | 0.0462 | 0.188~0.369 | 36.260 | <0.001 |
| Diagnosis*time (LMCI vs NC) | -0.436 | 0.0931 | -0.618~-0.253 | 21.920 | <0.001 |
| Diagnosis*time (EMCI vs NC) | -0.14 | 0.0802 | -0.297~0.017 | 3.043 | 0.081 |
| Digital forward |  |  |  |  |  |
| Intercept | 9.979 | 0.3498 | 9.293~10.665 | 813.988 | <0.001 |
| time | 0.004 | 0.0194 | -0.034~0.042 | 0.037 | 0.847 |
| Diagnosis*time (LMCI vs NC) | 0 | 0.03 | -0.059~0.058 | 0.000 | 0.989 |
| Diagnosis*time (EMCI vs NC) | -0.024 | 0.0316 | -0.086~0.038 | 0.579 | 0.447 |
| Digital backward |  |  |  |  |  |
| Intercept | 6.586 | 0.4419 | 5.720~7.453 | 222.130 | <0.001 |
| time | 0.06 | 0.0233 | 0.014~0.106 | 6.659 | 0.01 |
| Diagnosis*time (LMCI vs NC) | -0.086 | 0.0344 | -0.153~-0.018 | 6.182 | 0.013 |
| Diagnosis*time (EMCI vs NC) | -0.015 | 0.0346 | -0.082~0.053 | 0.176 | 0.675 |
| Trail A (sec) |  |  |  |  |  |
| Intercept | -5.567 | 5.5386 | -16.423~5.289 | 1.010 | 0.315 |
| time | -0.854 | 0.2552 | -1.355~-0.354 | 11.209 | 0.001 |
| Diagnosis*time (LMCI vs NC) | 2.027 | 0.4366 | 1.172~2.883 | 21.564 | <0.001 |
| Diagnosis*time (EMCI vs NC) | 0.132 | 0.3597 | -0.573~0.837 | 0.135 | 0.713 |
| Trail B (sec) |  |  |  |  |  |
| Intercept | -22.167 | 10.998 | -43.722~-0.611 | 4.062 | 0.044 |
| time | -3.607 | 0.6117 | -4.806~-2.408 | 34.760 | <0.001 |
| Diagnosis*time (LMCI vs NC) | 5.844 | 0.8634 | 4.152~7.536 | 45.814 | <0.001 |
| Diagnosis*time (EMCI vs NC) | 2.381 | 0.8887 | 0.639~4.123 | 7.179 | 0.007 |
| Verbal fluency |  |  |  |  |  |
| Intercept | 21.403 | 1.612 | 18.243~24.562 | 176.283 | <0.001 |
| time | 0.544 | 0.1178 | 0.313~0.775 | 21.340 | <0.001 |
| Diagnosis*time (LMCI vs NC) | -1.044 | 0.1461 | -1.330~-0.758 | 51.047 | <0.001 |
| Diagnosis*time (EMCI vs NC) | -0.421 | 0.1439 | -0703~-0.139 | 8.570 | 0.003 |

NC: normal cognition; EMCI: early mild cognitive impairment; LMCI: late mild cognitive impairment; GDS: Geriatric Depression Scale; MMSE: mini-mental status examination; STM: short-term memory; WMS-LM: Wechsler memory scale-logical memory; CVVLT: Chinese version of the verbal learning test; CFT: complex figure test; BNT: Boston naming test; TMT: trail making test.
