# Supplementary material for: The Clinical Course of Early and Late Mild Cognitive Impairment
Source: Front Neurol. 2022 May 16;13:685636. doi: 10.3389/fneur.2022.685636 (PMC9149311; doi:10.3389/fneur.2022.685636)
Supplement: Supplementary Table 2 — Generalized Estimating Equation (GEE) analysis of annual change rate differences between EMCI and LMCI in each neuropsychological test. NC, normal cognition; EMCI, early mild cognitive impairment; LMCI, late mild cognitive impairment; GDS, Geriatric Depression Scale; MMSE, mini-mental status examination; STM, short-term memory; WMS-LM, Wechsler memory scale-logical memory; CVVLT, Chinese version of the verbal learning test; CFT, complex figure test; BNT, Boston naming test; TMT, trail making test. [file Table_2.DOCX]

Supplementary: table 2 Generalized Estimating Equation (GEE) analysis of annual change rate differences between EMCI and LMCI in each neuropsychological test

| Variable | Regression coefficient | SE | 95% CI | χ 2 | p |
| --- | --- | --- | --- | --- | --- |
| MMSE | 1.926 | 0.2676 | 1.402~2.451 | 51.799 | <0.001 |
| STM | 0.715 | 0.0835 | 0.551~0.879 | 73.248 | <0.001 |
| WMS Logical memory | 4.656 | 0.3245 | 4.020~5.292 | 205.830 | <0.001 |
| CVVLT total recall | 3.988 | 0.4746 | 3.057~4.918 | 70.587 | <0.001 |
| CVVLT delayed recall | 2.430 | 0.2417 | 1.956~2.904 | 101.071 | <0.001 |
| CFT immediate recall | 4.733 | 0.7678 | 3.228~6.238 | 38.000 | <0.001 |
| CFT delayed recall | -6.283 | 0.1400 | -6.557~-6.009 | 2015.415 | <0.001 |
| CFT copy | 1.038 | 0.4245 | 0.206~1.870 | 5.982 | 0.014 |
| Clock drawing | 0.588 | 0.1837 | 0.220~0.948 | 10.325 | 0.001 |
| BNT | 1.025 | 0.2915 | 0.454~1.596 | 12.362 | <0.001 |
| Digital forward | 0.015 | 0.0931 | -0.168~0.197 | 0.025 | 0.874 |
| Digital backward | 0.250 | 0.0991 | 0.056~0.444 | 6.372 | 0.012 |
| Trail A (sec) | -6.889 | 1.4286 | -9.689~-4.089 | 23.257 | <0.001 |
| Trail B (sec) | 0.044 | 0.0269 | -0.009~0.096 | 2.653 | 0.103 |
| Verbal fluency | -12.523 | 2.659 | -17.73~-7.309 | 22.165 | <0.001 |

NC: normal cognition; EMCI: early mild cognitive impairment; LMCI: late mild cognitive impairment; GDS: Geriatric Depression Scale; MMSE: mini-mental status examination; STM: short-term memory; WMS-LM: Wechsler memory scale-logical memory; CVVLT: Chinese version of the verbal learning test; CFT: complex figure test; BNT: Boston naming test; TMT: trail making test.
